# Glycaemic Control Impact on Renal Endpoints in Diabetic Patients on Haemodialysis

**DOI:** 10.1155/2015/523521

**Published:** 2015-09-20

**Authors:** Danielle Creme, Kieran McCafferty

**Affiliations:** Royal London Hospital, Whitechapel Road, London E1 1BB, UK

## Abstract

*Objective*. To identify the number of haemodialysis patients with diabetes in a large NHS Trust, their current glycaemic control, and the impact on other renal specific outcomes.* Design*. Retrospective, observational, cross-sectional study.* Methods*. Data was collected from an electronic patient management system. Glycaemic control was assessed from HbA1c results that were then further adjusted for albumin (Alb) and haemoglobin (Hb). Interdialytic weight gains were analysed from weights recorded before and after dialysis, 2 weeks before and after the most recent HbA1c date. Amputations were identified from electronic records.* Results*. 39% of patients had poor glycaemic control (HbA1c > 8%). Adjusted HbA1c resulted in a greater number of patients with poor control (55%). Significant correlations were found with interdialytic weight gains (*P* < 0.02, *r* = 0.14), predialysis sodium (*P* < 0.0001, *r* = −1.9), and predialysis bicarbonate (*P* < 0.02, *r* = 0.12). Trends were observed with albumin and C-reactive protein. Patients with diabetes had more amputations (24 versus 2).* Conclusion*. Large number of diabetic patients on haemdialysis have poor glycaemic control. This may lead to higher interdialytic weight gains, larger sodium and bicarbonate shifts, increased number of amputations, and possibly increased inflammation and decreased nutritional status. Comprehensive guidelines and more accurate long-term tests for glycaemic control are needed.

## 1. Introduction

Diabetes and renal disease are both long-term conditions prevalent in the adult UK population at 14% and 7.5%, respectively [1]. The single most common aetiology of CKD is diabetic nephropathy. It is estimated that a quarter of all people with diabetes will develop end stage renal failure (ESRF), causing 21% and 11% of all deaths in type 1 and type 2 diabetes, respectively [2].

For people with diabetes requiring dialysis, one-year mortality rate was 17% compared with 11% for nondiabetic dialysis patients [3]. Other factors influence mortality such as old age, male gender, South Asian ethnicity, and lower socioeconomic status [4].

Slowing progression to ESRF in this patient group is the main treatment strategy for patients with diabetic nephropathy. While there is a plethora of research and evidence on methods and treatment to achieve this, outcomes and care once RRT (renal replacement therapy) has been initiated are less well defined.

It has been suggested that this patient group has a higher risk of complications such as cardiovascular disease, vascular calcification, infection, lower limb amputation, peripheral neuropathy, retinopathy, difficulties with vascular access, depression, and generalized decrease in quality of life (QoL) [5]. The severity of these complications could be mediated with better glycaemic control even after RRT initiation. 

*Aims*. Aims of the study were to assess the prevalence of diabetes in the haemodialysis population to categorise the glycaemic control of the HD population into high and low risk categories and investigate the impact of poor glycaemic control on other renal specific outcomes.

## 2. Methods

Ethical approval was provided by the Clinical Effectiveness Unit and Audit Unit at Barts Health NHS Trust (registration number 5413). All patients on haemodialysis (HD) in August 2014 were included. Data was extracted from electronic data records (Renalware).

Inclusion criteria were patients >18 years, currently on HD at Barts Health NHS Trust. Patients without diabetes or an HbA1c were excluded from the biochemical analysis.

The most recent recorded HbA1c was used to quantify glycaemic control. The patients were then stratified into HbA1c ranges to assess glycaemic control. Stratification was based on available published evidence. All other biochemistry and amputation data was extracted from Renalware at the time of the individuals HbA1c date.

Interdialytic weight gains (IDWG) were calculated using weights before and after dialysis, 2 weeks before and after HbA1c date. Nonparametric data was analyzed using the Kruskal-Wallis test statistic with Dunn's postcomparison test while correlations were quantified using the Spearman correlation coefficients with GraphPad Prism 6 software (San Diego, CA, USA).

HbA1c was further adjusted for HD accounting for changes in albumin (Alb) and haemoglobin (Hb)

(i) AG^*∗*^ = 104.8 + 29.7 × HbA1c − 18.4 × Alb − 4.7 × Hb [6]

^*∗*^Average glucose.

## 3. Results

Out of 979 patients receiving HD at the time, 42% had Diabetes Mellitus (DM). Total number of diabetic patients receiving HD, *n* = 412 (42% of all HD patients). There was a significantly higher number of South Asians and older people with diabetes compared to the nondiabetic HD cohort (*p* < 0.0001) (Table 1).

39% of patients Trust wide had poorly controlled diabetes defined as HbA1c <5.4% (36 mmol/mol) or >8% (64 mmol/mol). 7% of these were at high risk defined as HbA1c >10% (86 mmol/mol).

Significant differences were seen with various biochemical and clinical parameters (ALP, Hb, sodium, and pre-HD systolic BP) when comparing each HbA1c category (Table 2). However, when each at-risk category (HbA1c outside the target range of 5.4%–7.9%) was compared to the optimally controlled group (HbA1c 5.4%–7.9%) using Dunn's multiple comparison testing, there were no significant differences seen between the groups.

**Figure 1:**
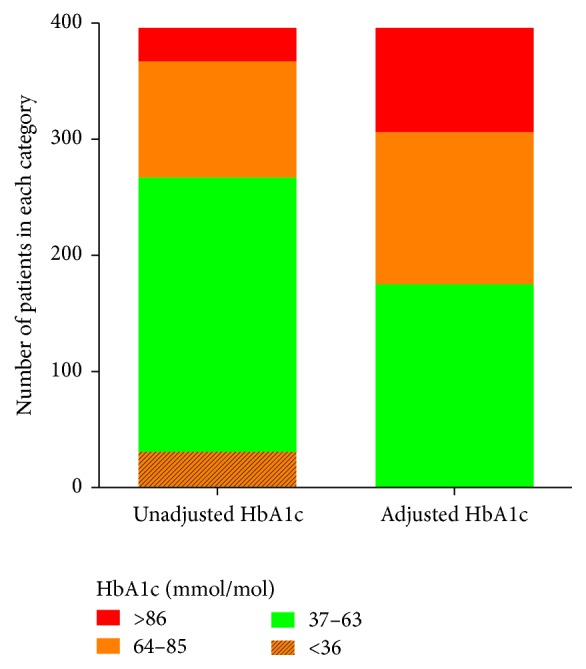
Glycaemic control assessment using HbA1c adjusted for albumin and haemoglobin.

There was an increase of 16% of patients with poorly controlled diabetes with HbA1c adjusted for Alb and Hb (Figure 1).

### 3.1. Correlations

**Figure 2:**
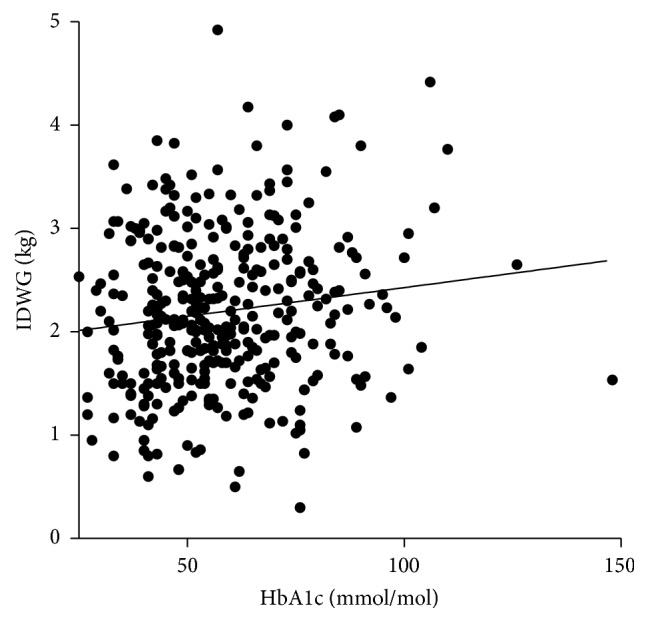
Correlation between glycaemic control and interdialytic weight gains.

**Figure 3:**
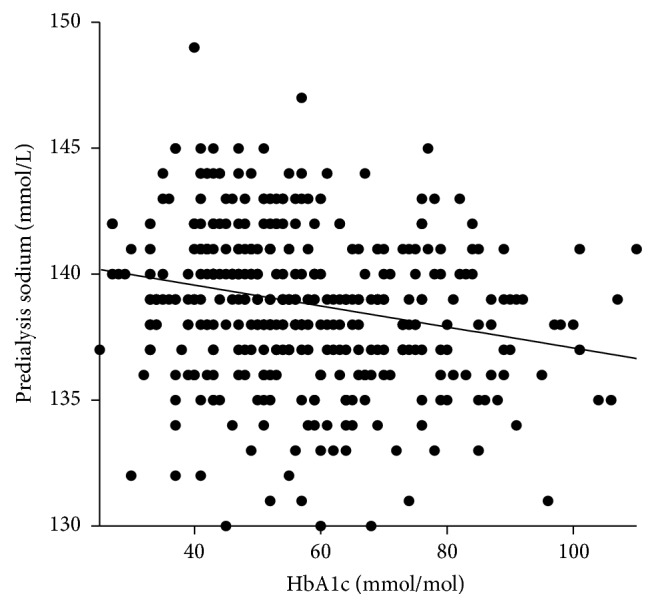
Correlation between glycaemic control and predialysis sodium.

**Figure 4:**
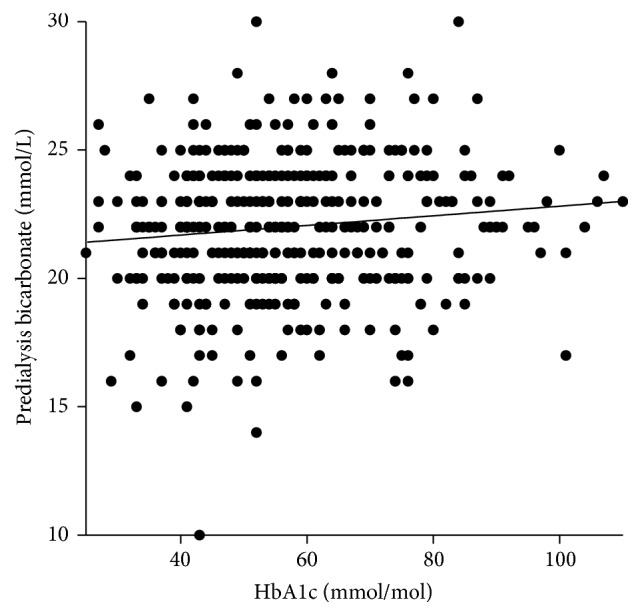
Correlation between glycaemic control and predialysis bicarbonate.

Higher HbA1c is associated with larger fluid gains as there is a small (*r* = 0.14) but statistically and clinically significant (*p* < 0.02) positive correlation between IDWG and poorly controlled diabetes (Figure 2). There is a significant increase in systolic blood pressure with higher HbA1c values. There is a weak significant (*p* < 0.0001) negative correlation (*r* = −1.9) between poor glycaemic control and predialysis sodium (Figure 3). There is a weak (*r* = 0.12) but statistically significant (*p* < 0.02) correlation between poor glycaemic control and an increase in bicarbonate (Figure 4). There is a trend between poor glycaemic control, raised CRP, and reduced Alb which is not significant (*p* = 0.07 and *p* = 0.08, resp.). The proportion of patients with amputation who have diabetes is greater than those who do not, with 24 versus 2 patients, respectively, with 46% (11 patients) having an HbA1c >8% (65 mmol/mol). No other correlations between poor glycaemic control and other biochemical markers were observed (haemoglobin, ferritin, TSAT, WCC, potassium, phosphate, PTH, and vitamin D).

## 4. Discussion

There is scant information published nationally or internationally on glycaemic control in HD-dependent diabetic patients. The renal registry has no data on glycaemic control of RRT patients.

### 4.1. Assessment of Glycaemic Control

There are several challenges in accurately assessing glycaemic control in this patient group. In the diabetic population without diabetic nephropathy and before RRT, HbA1c test is the gold standard test used to assess long-term glycaemic control. An HbA1c test will reflect the average amount of glucose a red blood cell (RBC) was exposed to during its life span.

There are several issues affecting the accuracy of HbA1c tests with ESRF. Urea derived isocyanate results in carbamylated Hb which is indistinguishable from glycated Hb, giving a false elevation in readings. Other inaccuracies arise from disruptions to RBC life span on which the test is based. Iron deficiency, B_12_, or folate deficiencies also give falsely high readings as they extend RBC life span. Reduced RBC life span as a consequence of dialysis, recent transfusions and accelerated erythropoiesis result in falsely low results due to shorter periods of exposure to glucose.

Other tests are available such as glycated albumin or glycated fructosamine. These reflect control over a shorter time period and are less affected by the unstable haemodynamic environment as HbA1c. Other factors such as increased protein turnover and malnutrition can affect the accuracy and validity of these tests. Their advantages and disadvantages have been discussed in several papers [7–9]. There are scant long-term trials looking at glycated albumin and chronic complications of diabetes, although one small long-term study found that glycated albumin correlated well with cardiovascular mortality [10]. A consensus on the best methods for assessing long-term glycaemic control in this patient cohort has yet to be reached. Clearly, there is a need for longer, more extensive research in this area. While there are good arguments for using these tests over HbA1c, at present, HbA1c remains the test used most often to monitor and determine glucose control in HD patients.

### 4.2. Adjusted HbA1c

A recent large study (*n* = 11,986) [6], having recognized anaemia, malnutrition, and inflammation's impact on survival in this patient cohort, has developed equation models adjusting for these confounding factors including Alb (model 3, *R* ^2^ = 0.483), Alb + Hb (model 4, *R* ^2^ = 0.486), and Alb + Hb + age + race (model 5, *R* ^2^ = 0.491). These all showed a stronger association than the DCCT A_1c_ derived average glucose equation did (*R* ^2^ = 0.468) with daily blood glucose. As this study was based in the USA, where ethnicity is very different to the present cohort, we applied model 4 to the data.

Following this adjustment, the number of patients with poor glycaemic control rose from 39% to 55%, eliminating those who appeared to have very low HbA1c. This highlights the concern of using tests which are inaccurate in this patient cohort. Using standard HbA1c results misclassifies patients into lower risk categories; care therefore is not being efficiently or effectively targeted.

The most accurate method of assessing glucose control is regular self-monitoring of plasma glucose with finger prick tests. Target glucose levels for diabetic patients on HD suggested by one study were fasting <7.8 mmol/L and postprandial <11.1 mmol/L [11], giving an average HbA1c reading of <9%. Compliance and adherence are issues with this method as they involve regular pre- and postprandial daily tests which some patients find difficult to manage.

### 4.3. HbA1c Targets

While target HbA1c levels for diabetic population without CKD or ESRF are clearly defined based on large-scale studies (DCCT/EDIC, ACCORD, ADVANCE, and VADT), a consensus on HbA1c levels for the diabetic population receiving RRT has not been established. Current advice is centered around the prevention of hyperglycaemia and microvascular complications of diabetes.

- *Dialysis Outcomes and Practice Patterns Study (DOPPS)*. It is 7%-8% (53–64 mmol/mol), suggesting lower end for younger patients with fewer comorbidities and higher end for older patients with greater number of comorbidities [12].
- *Kidney Disease Outcomes Quality Initiative (KDOQI)*. CKD population is 7% (53 mmol/mol), although in their rationale they mention that HbA1c levels of 7–9% are associated with better outcomes for survival, hospitalization, and CVD in patients on haemodialysis in most but not all observational studies [13].
- *Renal association. *It is <7.5% (<58 mmol/mol) [14].

Using survival and cardiovascular mortality as outcomes, several studies have attempted to stratify optimal and suboptimal glucose control. The most recent meta-analysis of observational studies suggested a J shaped curve relationship where an increase in mortality was associated with HbA1c <5.4% and >8.5% [15].

Ricks et al. [16], looking at >54,000 patient, also found increased mortality risks with HbA1c below and above 7%–7.9%, with <5% yielding hazard ratio of 1.35, >8% HR of 1.06 and >10% HR of 1.19.

There are no published national data looking at glycaemic control for diabetic patients on HD; therefore, it is difficult to assess whether the glycaemic control of the present cohort is better or worse than others. Irrespective, poor control in over half of the patients indicates that more directed and effective care is needed.

As noted by O'Toole et al. [17], besides the inherent problems with using HbA1c as a measure of glycaemic control in this population, there are several further confounding factors that must be considered when using HbA1c levels as a target outcome. Patients with very poor glycaemic control at the outset of dialysis are likely to have worse outcomes. In addition, poor glycaemic control is a surrogate for other factors contributing to poor self-care such as smoking and reduced adherence with medication, fluid restriction, and dietary recommendations.

### 4.4. Correlations and Additional Complications

Fluid management is one of the main challenges in HD with increased complexity in diabetic patients. Hyperglycaemia triggers osmoreceptors to stimulate thirst, leading to fluid consumption. Large IDWG and fluid overload lead to volume expansion and cardiac hypertrophy [18, 19] exacerbating pulmonary and cardiovascular symptoms. There are several methods for assessing IDWG, either in kilograms calculated from postdialysis weight to the following predialysis weight or by percentage weight change from dry weight. There are positives and negatives for each method; the first while being easier to calculate does not include dry weight in its estimation, while the second, allowing for proportional fluid to body mass estimations, relies on an estimated dry weight which may also be inaccurate.

In our study, we defined undesirable IDWG according to the KDOQI rationale of >2.5 kg between dialysis sessions [20] and observed a clinically and statistically significant (*p* < 0.02) if weak (*r* = 0.14) correlation between glycaemic control and IDWG (Figure 2). A recent prospective, 3-year follow-up study looking at >10,000 patients, found an increase in all cause and cardiovascular mortality rate in patients who had >2 kg IDWG from target weight in >30% of dialysis sessions [21]. The effect of glycaemia on IDWG was also reported by Davenport [22] who noted absolute and percentage IDWG was the lowest in the group with the best diabetic control classified as <6% versus poor control at >8% (2.0 ± 1 kg and 2.76 ± 1.5% versus 2.5 ± 1.1 kg and 3.3 ± 1.3% (*p* < 0.05), resp.). Our study supports the association of poor glycaemic control with excessive fluid intake; therefore, in a diabetic cohort where cardiovascular disease is already more prevalent, it is pertinent to prevent further exacerbation by undesirable IDWG by attempting to control hyperglycaemia.

Recent studies have elucidated an association between low predialysis Na and all cause and cardiovascular mortality [23, 24]. A further recent paper that looked at the relationship between predialysis Na levels, IDWG, and nutritional status found that low Na (<136.2 mlEq/L) was associated with increased IDWG and decreased lean body mass [25]. Our study shows that hyperglycaemia may exacerbate low Na as revealed, a statistically significant association between elevated HbA1c and low Na (*p* < 0.0001) (Figure 3). Predialysis sodium decreases with worsening glycaemic control due to translocational hyponatraemia. In marked hyperglycemia, extracellular fluid (ECF) osmolality rises and exceeds that of intracellular fluid (ICF). Glucose penetrates cell membranes slowly in the absence of insulin, resulting in movement of water out of cells into the ECF. Serum Na^+^ concentration falls in proportion to the dilution of the ECF and therefore poor glycaemic control may be an additional contributing factor to hyponatraemia, increased mortality risk, high IDWG, and malnutrition.

The statistically significant correlation with elevated bicarbonate levels and raised HbA1c (*p* < 0.002) (Figure 4) was more difficult to explain, as hyperglycaemia usually increases acidosis due to hyperosmolality and release of free fatty acids and ketones, resulting in reduced pH; as such one would expect a lower bicarbonate level with poor glycaemic control. One explanation may be that patients with well controlled glycaemia are better nourished, choosing protein rich foods over carbohydrates. The increased protein intake may result in increased systemic acidity, therefore giving an inverse bicarbonate relationship. A recent paper looking at pH and bicarbonate association with mortality in HD patients noted that predialysis elevated pH was associated with increased risk of mortality but not before or after bicarbonate [26] which may render the association observed clinically insignificant.

Many observational studies have noted that HD patients are chronically inflamed. Insulin resistance and diabetes are also known to increase inflammatory cytokines although the precise mechanism remains unclear. In addition, there may be a reciprocal relationship present where inflammation and infection precipitate hyperglycaemia which in turn exacerbates and prolongs the inflammatory response. Hyperglycaemia is a known driving force for ischemia and poor wound healing which may also explain the far greater number of patients with amputations in the diabetic versus nondiabetic population, with 46% of patients with amputations having an HbA1c >8% (65 mmol/mol). A recent meta-analysis corroborated the increased risk of foot ischemia and lower limb amputation with higher HbA1c values [27].

While not a hard outcome like mortality, amputations have a major impact on a patient's QoL. A small interventional study (*n* = 83) by McMurray et al. [28] found that intensive glycaemic control intervention resulted in decreased need for amputations and hospitalisation and increase in QoL.

There may be a relationship between hyponatraemia, inflammation, and nutritional status in HD patients, which the study by Poulikakos et al. [25] tried to explore, although, as with our own study, the associations with CRP and albumin, while showing a positive trend, were not statistically significant.

The strengths of this paper lie in the attempt to stratify diabetic patients on HD into risk categories and observe impact on renal specific clinical parameters such as electrolyte imbalances and IDWG, which has not been done previously in the UK. Study design is a limitation as cross-sectional observational studies cannot prove causative effects and the reliance on a single measure of HbA1c to quantify glycaemic control may not be an accurate representation of a patient's long-term glycaemic control.

Nevertheless, while most studies focus on hard outcomes such as mortality, there are many other factors in the patient's journey which are important. Achieving euglycaemia in patients with diabetes in the context of ESRD and HD is complex and multifactorial; however, it is essential for improving their clinical progress and QoL.

## 5. Conclusion

Glycaemic control in many HD dependent patients with diabetes is poor and may lead to additional complications such as high IDWG, electrolyte imbalance, and amputations. Current tests for long-term glycaemic control are inaccurate and may result in misclassification of patients into lower risk categories leading to misdirected management. There is an urgent need for further research to provide more accurate tests for long-term monitoring of glycaemic control and long-term prospective studies into interventions for alternative outcomes such as improvement with amputations, inflammation, undesirable IDWG, nutritional status, and QoL.

## Figures and Tables

**Table 1:** Demographics.

	Diabetes present (*n* = 412)	No diabetes present (*n* = 567)
Ethnicity *n* (%)		
White	90 (22)	203 (36)
Black	114 (28)	176 (31)
South Asian	179 (43)	125 (22)
Other	29 (7)	63 (11)

Age (years)	65 (57–73)	56 (45–70)
Dialysis vintage (years)	2.6 (1.3–4.9)	2.9 (1.1–5.5)
Male gender *n* (%)	228 (55)	334 (59)

*Diabetic cohort only*		
Type of diabetes		
Type 1 *n* (%)	15 (4)	
Type 2 *n* (%)	397 (96)	
Insulin treated *n* (%)	260 (63)	
Kt/V	1.5 (1.4–1.7)	

**Table 2:** Comparison of biochemistry of diabetic cohort according to glycaemic control.

Variable	<5.4%	5.4–7.9%	8–9.9%	>10%	*p* value
	<36 mmol/mol	37–63 mmol/mol	64–85 mmol/mol	>86 mmol/mol	
Urea (mmol/L)	18.5 (10.8–21.5)	16.8 (13.2–22.9)	17.5 (12.8–21.3)	17.5 (9.4–23.8)	0.98
Creatinine (*μ*mol/L)	682 (570–839)	706 (571–845)	710 (558–853)	728 (624–854)	0.77
Albumin (g/L)	40 (37–43)	40 (38–42)	41 (38–43)	40 (37–41)	0.09
CRP (mg/L)	6 (5–15)	6 (5–20)	6 (5–14)	6 (5–17)	0.87
Hb (g/dL)	10.3 (8.9–11.2)	10.6 (9.6–11.5)	10.9 (10–11.7)	11 (9.8–11.9)	0.02
Ferritin (mcg/L)	394 (195–571)	445 (279–623)	458 (291–647)	496 (277–837)	0.69
TSAT (%)	22 (17–29)	25 (21–32)	26 (21–34)	26 (21–35)	0.2
Calcium (mmol/L)	2.3 (2.2–2.4)	2.3 (2.2–2.4)	2.3 (2.2–2.4)	2.2 (2.1–2.3)	0.06
Phosphate (mmol/L)	1.5 (1.3–1.9)	1.4 (1.1–1.7)	1.5 (1.2–1.9)	1.5 (1.3–1.9)	0.2
PTH (pmol/L)	44 (36–91)	40 (20–52)	34 (16–57)	41 (32–65)	0.1
ALP (unit/L)	99 (64–138)	117 (85–162)	104 (80–145)	134 (91–224)	0.01
Sodium (mmol/L)	139 (138–141)	138 (136–140)	139 (137–141)	138 (135–140)	0.002
Bicarbonate (mmol/L)	22 (20–23)	22 (20–24)	22 (20–24)	22 (22–24)	0.4
Potassium (mmol/L)	5.1 (4.6–5.7)	4.8 (4.5–5.5)	4.9 (4.4–5.4)	4.8 (4.5–5.5)	0.5
Kt/V	1.4 (1.4–1.8)	1.5 (1.3–1.7)	1.6 (1.4–1.7)	1.4 (1.4–1.6)	0.61
Pre-HD systolic BP (mm/Hg)	141 (131–159)	158 (138–175)	150 (132–170)	179 (133–202)	0.02
